# Health and happiness: cross-sectional household surveys in Finland, Poland and Spain

**DOI:** 10.2471/BLT.13.129254

**Published:** 2014-08-13

**Authors:** Marta Miret, Francisco Félix Caballero, Somnath Chatterji, Beatriz Olaya, Beata Tobiasz-Adamczyk, Seppo Koskinen, Matilde Leonardi, Josep Maria Haro, José Luis Ayuso-Mateos

**Affiliations:** a Instituto de Salud Carlos III, Centro de Investigación Biomédica en Red de Salud Mental (CIBERSAM), Madrid, Spain.; b Department of Health Statistics and Information Systems, World Health Organization, Geneva, Switzerland.; c CIBERSAM, Parc Sanitari Sant Joan de Déu, Barcelona, Spain.; d Department of Medical Sociology, Jagiellonian University Medical College, Krakow, Poland.; e National Institute for Health and Welfare, Helsinki, Finland.; f Fondazione IRCCS, Neurological Institute Carlo Besta, Milan, Italy.; g Department of Psychiatry, Universidad Autónoma de Madrid, CIBERSAM, C/ Arzobispo Morcillo 4, 28029, Madrid, Spain.

## Abstract

**Objective:**

To explore the associations between health and how people evaluate and experience their lives.

**Methods:**

We analysed data from nationally-representative household surveys originally conducted in 2011–2012 in Finland, Poland and Spain. These surveys provided information on 10 800 adults, for whom experienced well-being was measured using the Day Reconstruction Method and evaluative well-being was measured with the Cantril Self-Anchoring Striving Scale. Health status was assessed by questions in eight domains including mobility and self-care. We used multiple linear regression, structural equation models and multiple indicators/multiple causes models to explore factors associated with experienced and evaluative well-being.

**Findings:**

The multiple indicator/multiple causes model conducted over the pooled sample showed that respondents with younger age (effect size, *β* = 0.19), with higher levels of education (*β* = −0.12), a history of depression (*β* = −0.17), poor health status (*β* = 0.29) or poor cognitive functioning (*β* = 0.09) reported worse experienced well-being. Additional factors associated with worse evaluative well-being were male sex (*β* = −0.03), not living with a partner (*β* = 0.07), and lower occupational (*β* = −0.07) or income levels (*β* = 0.08). Health status was the factor most strongly correlated with both experienced and evaluative well-being, even after controlling for a history of depression, age, income and other sociodemographic variables.

**Conclusion:**

Health status is an important correlate of well-being. Therefore, strategies to improve population health would also improve people’s well-being.

## Résumé

**Objectif:**

Étudier les associations entre la santé et la manière dont les personnes évaluent et ressentent leur vie.

**Méthodes:**

Nous avons analysé les données tirées des enquêtes sur les ménages représentatifs à l'échelle nationale qui ont été menées en 2011–2012 en Finlande, Pologne et Espagne. Ces enquêtes ont fourni des informations sur 10 800 adultes, dont le bien-être a été mesuré en utilisant la méthode de reconstruction de la journée et le bien-être évaluatif a été quantifié avec l'échelle de Cantril (Cantril Self-Anchoring Striving Scale). L'état de santé a été évalué par des questions dans huit domaines, y compris la mobilité et le fait de prendre soin de soi-même. Nous avons utilisé des modèles d'équation structurelle à régression linéaire multiple et des modèles à indicateurs et à causes multiples pour étudier les facteurs associés au bien-être ressenti et évaluatif.

**Résultats:**

Le modèle à indicateurs et à causes multiples appliqué sur l'échantillon groupé a montré que les répondants jeunes (ampleur de l'effet [*β*]  =  0,19), avec un niveau d'éducation plus élevé (*β* = −0,12), des antécédents de dépression (*β* = −0,17), un mauvais état de santé (*β* = 0,29) ou de faibles facultés cognitives (*β* = 0,09) avaient signalé le plus faible bien-être ressenti. Des facteurs supplémentaires associés avec la plus mauvaise évaluation de bien-être étaient: être de sexe mâle (*β* = −0,03), ne pas vivre avec un partenaire (*β* = 0,07), un statut professionnel inférieur (*β* = −0,07) ou des niveaux de revenus plus faibles (*β* = 0,08). L'état de santé était le facteur le plus corrélé à la fois avec le bien-être ressenti et évaluatif, même après contrôle des antécédents de dépression, de l'âge, des revenus et des autres variables sociodémographiques.

**Conclusion:**

L'état de santé est un facteur de corrélation important du bien-être. Par conséquent, les stratégies d'amélioration de la santé de la population devraient également améliorer le bien-être des personnes.

## Resumen

**Objetivo:**

Examinar la relación entre la salud y cómo las personas evalúan y experimentan sus vidas.

**Métodos:**

Se analizaron datos de encuestas a hogares representativos a nivel nacional realizadas inicialmente entre 2011 y 2012 en Finlandia, Polonia y España. Estas encuestas proporcionaron información sobre 10 800 adultos, para quienes el bienestar experiencial se midió con el método de reconstrucción de día y el bienestar evaluativo se midió con la escala Cantril. Se evaluó el estado de salud mediante preguntas de ocho ámbitos, que incluían la movilidad y el cuidado personal. Se emplearon modelos de regresión lineal múltiple y ecuaciones estructurales, así como modelos de causas múltiples/indicadores múltiples a fin de examinar los factores asociados con el bienestar experiencial y evaluativo.

**Resultados:**

El modelo de causas múltiples/indicadores múltiples realizado en la muestra combinada mostró que los encuestados de menor edad (tamaño del efecto [*β*] = 0,19), con niveles superiores de educación (*β* = −0,12), antecedentes de depresión (*β* = −0,17), mal estado de salud (*β* = 0,29) o mal funcionamiento cognitivo (*β* = 0,09) presentaron menor bienestar experiencial. Otros factores asociados con menor bienestar evaluativofueron el sexo masculino (*β* = −0,03), no vivir en pareja (*β* = 0,07) y un nivel ocupacional *β* = −0,07) o de ingresos inferior (*β* = 0,08). El estado de salud fue el factor que más se correlacionó tanto con el bienestar experiencial como evaluativo, incluso después de examinar los antecedentes de depresión, la edad, los ingresos y otras variables sociodemográficas.

**Conclusión:**

El estado de salud guarda una correlación importante con el bienestar. Por lo tanto, las estrategias para mejorar la salud de la población podrían asimismo mejorar el bienestar de las personas.

## ملخص

**الغرض:**

دراسة الارتباطات بين الصحة وطريقة تقييم الأشخاص لحياتهم وخبراتهم الحياتية.

**الطريقة:**

قمنا بتحليل البيانات المستمدة من دراسات استقصائية ذات عينات ممثلة على الصعيد الوطني أجريت في الأصل في عامي 2011 و2012 في فنلندا وبولندا وأسبانيا. وقدمت هذه الدراسات الاستقصائية معلومات عن 10800 شخص بالغ، تم قياس مستوى الرفاهية لديهم باستخدام طريقة إعادة بناء اليوم وتم قياس مستوى الرفاهية التقييمي باستخدام مقياس كانتريل للتحديد الذاتي للازدهار. وتم تقييم الحالة الصحية عن طريق طرح أسئلة في ثمانية مجالات شملت الانتقال والرعاية الذاتية. واستخدمنا الارتداد الخطي المتعدد ونماذج المعادلة الهيكلية ونماذج المؤشرات المتعددة/ الأسباب المتعددة لاستكشاف العوامل المرتبطة بمستوى الرفاهية الفعلي والتقييمي.

**النتائج:**

أظهر نموذج المؤشرات المتعددة/ الأسباب المتعددة الذي أجري على العينة المجمعة أن المشاركين صغار السن (حجم الأثر [معامل بيتا = 0.19)، الذين يتمتعون بمستويات تعليم عالية (معامل بيتا = -0.12)، أو لديهم تاريخ من الاكتئاب (معامل بيتا = -0.17) أو يعانون من سوء الحالة الصحية (معامل بيتا = 0.29) أو سوء الوظائف الإدراكية (معامل بيتا = 0.09) أبلغوا عن مستوى رفاهية أسوأ. وكانت العوامل الإضافية المرتبطة بمستوى الرفاهية التقييمي الأسوأ هي ذكورية النوع (معامل بيتا = -0.03) وعدم العيش مع شريك (معامل بيتا = 0.07) وانخفاض المستوى المهني (معامل بيتا = -0.07) أو مستوى الدخل (معامل بيتا = 0.08). وكانت الحالة الصحية العامل الأقوى ارتباطاً بكل من مستوى الرفاهية الفعلي والتقييمي، حتى بعد ضبط متغيرات تاريخ الاكتئاب والسن والدخل وغيرها من المتغيرات الاجتماعية الديمغرافية.

**الاستنتاج:**

تمثل الحالة الصحية أحد العوامل المهمة المرتبطة بمستوى الرفاهية. ومن ثم، ستؤدي الاستراتيجيات الرامية إلى تحسين صحة السكان إلى تحسين مستوى رفاهية الأشخاص كذلك.

## 摘要

**目的:**

探索健康与人们如何评价和体验其生活之间的关联。

**方法:**

我们分析了2011-2012年最初在芬兰、波兰和西班牙进行的国家横断面家庭调查的数据。这些调查提供了10800名成年人的信息，对其使用昨日重现法（Day Reconstruction Method）衡量幸福体验，并使用坎特里尔自我定位奋斗量表（Cantril Self-Anchoring Striving Scale）衡量可评价的幸福。健康状态通过包括流动性和自我保健等八个领域的问题进行评估。我们使用多元线性回归、结构方程模型和多指标/多原因模型探索与体验和可评价幸福感相关的因素。

**结果:**

混合样本中执行的多指标/多原因模型显示，更年轻（效应量[*β*] = 0.19）、教育水平更高（*β*  = –0.12）、抑郁史（*β*  = –0.17）、不良健康状况（*β *= 0.29）或不良认知功能（*β *= 0.09）的受访者报告的幸福体验更差。与更差的可评价幸福相关的其他因素有男性（*β *= –0.03）、没有和家长一起生活（*β *= 0.07）和更低的就业（*β *= –0.07）或收入水平（*β *= 0.08）。健康状况是与体验和可评价幸福相关性最大的因素，甚至在控制了抑郁史、年龄、收入和其他社会人口变量之后也是如此。

**结论:**

健康状况是幸福感的重要关联因素。因此，改善人口健康的策略也将改善民生幸福度。

## Резюме

**Цель:**

Изучить связь между здоровьем и тем, как люди оценивают и воспринимают свою жизнь.

**Методы:**

Мы проанализировали данные национальных репрезентативных обследований домохозяйств, первоначально проведенных в 2011—2012 годах в Финляндии, Польше и Испании. Эти обследования представили информацию о 10 800 взрослых людях, степень фактического благополучия жизни которых измерялась с использованием метода реконструкции дня, а степень оценочного благополучия — на основе шкалы самооценки Кантрила. Состояние здоровья оценивалось по ответам на вопросы в восьми областях, включая мобильность и способность к самообслуживанию. Мы использовали модели множественной линейной регрессии, модели структурных уравнений и модели множественных индикаторов/множественных причин для изучения факторов, связанных с фактическим и оценочным благополучием.

**Результаты:**

Применение модели множественных индикаторов/множественных причин по совокупной выборке показало, что респонденты в более молодом возрасте (величина эффекта [*β*] = 0,19), с более высоким уровнем образования (*β* = –012), ранее страдавшие от депрессии (*β* =–0.17), с плохим состоянием здоровья (*β* = 0,29) или плохими когнитивными функциями (*β* = 0,09) сообщали о худшем оценочном благополучии, по сравнению с другими группами. Дополнительными факторами, связанными с худшим, по сравнению с другими группами, оценочным благополучием, являлись мужской пол (*β* =–0,03), проживание без партнера (*β*= 0,07) и более низкий уровень квалификации (*β*= –0.07) или доходов (*β*= 0,08). Состояние здоровья являлось фактором, наиболее сильно коррелирующим как с фактическим, так и с оценочным благополучием, даже после учета факторов депрессии, возраста, дохода и других социально-демографических переменных.

**Вывод:**

Состояние здоровья является важным фактором благополучия. Поэтому стратегии по улучшению здоровья населения также ведут к улучшению его благополучия.

## Introduction

Many national surveys are in progress to evaluate well-being as an indicator of societal progress that goes beyond traditional indices, such as gross domestic product (GDP). These surveys inform policy-makers about the factors that can affect the well-being of populations.^1^ The Commission on the Measurement of Economic Performance and Social Progress recommended shifting emphasis from measuring economic production to measuring people’s well-being and that this measurement be done at a national level.^2^ In line with these recommendations, the Better Life Initiative, launched by the Organisation for Economic Co-operation and Development, aims to measure society’s progress across eleven domains of well-being, such as life satisfaction, health, education and environment.^3^ Efforts are also being made at the national level in many countries.^4^^–^^9^

Health and well-being are interconnected, with well-being influencing health^10^^,^^11^ and health influencing well-being.^12^^,^^13^ Health is an important determinant of subjective well-being, together with other elements such as having a job, a partner and social contact.^13^ Good health is linked with greater well-being; while setbacks in health, such as serious diseases or disability, have negative effects on well-being.^12^

Research distinguishes between two different ways of assessing well-being. The first is to ask people to evaluate their life. The result is called evaluative well-being. The second way is to ask people to report the positive and negative emotions that they experience day-to-day. This is called experienced well-being. Evaluative well-being thus refers to a person’s overall evaluation of the quality of his or her life, whereas experienced well-being captures the positive and negative emotions that people experience from moment to moment.^14^ Assessing both dimensions is relevant, because these do not necessarily have the same correlates. For example, people with high income report more satisfaction with their lives when their evaluative well-being is assessed but these same people do not report better experienced well-being. Other life circumstances, such as marital status and education, are also more strongly correlated with evaluative than experienced well-being.^14^^,^^15^ On the other hand, ill health, caring for an adult, loneliness and smoking have been reported to be strong predictors of low experienced well-being.^14^ However, analysis of the correlation between health status and evaluative and experienced well-being has not been done at a population level. Therefore, we explored associations between health and evaluative and experienced well-being in three countries, and we tried to understand which part of the population has the highest risk of poor well-being.

## Methods

### Study design and data source

The Collaborative Research on Ageing in Europe project^16^ is a cross-sectional household survey of a probabilistic sample representative of the non-institutionalized adult population of Finland, Poland and Spain. We selected these countries to give a broad representation of European populations, health characteristics and welfare systems.^17^

The information was collected with a face-to-face structured interview carried out at respondents’ homes, via Computer-Assisted Personal Interviewing, between 8 April 2011 and 8 May 2012. The interviewers had participated in a training course for the administration of the survey. The questionnaires were based on the ones used in the World Health Organization (WHO) Study on Global Ageing and Adult Health (SAGE)^18^ and they were translated from English into Finnish, Polish and Spanish following the World Health Organization translation guidelines for assessment instruments.^19^ The translated questionnaires were piloted in 2010 in the countries and based on the feedback from the interviewers some changes and corrections were made. Quality assurance procedures were implemented during fieldwork.^20^

A multistage clustered design was used to obtain nationally representative samples. In Poland and Spain, a stratified multistage random sampling method was used and strata were created according to the geographical administrative regions and number of people living in the habitat. Age strata were used to select households according to the age structure of the population. The respondents were randomly selected among inhabitants of a household from a certain age group. In Finland, the design was a stratified two-stage cluster sampling design, and strata were created based on the largest towns and university hospital regions. A systematic sampling of people was conducted so that the sample size in each stratum was proportional to the corresponding population base.

A total of 10 800 individuals participated: 1976 from Finland, 4071 from Poland and 4753 from Spain. The countries’ response rates were 53.4%, 66.5% and 69.9% respectively.

### Key variables

We assessed experienced well-being with an abbreviated version of the Day Reconstruction Method,^21^ owing to its application in general population surveys.^22^^,^^23^ Participants reconstructed a portion of their previous day’s activities and reported the extent to which they experienced various emotions on a seven-point response scale ranging from 0 (not at all) to 6 (very much). Positive affect was defined as the average of the positive emotions (calm/relaxed and enjoying), weighted by the duration of the activities, with higher values indicating higher positive affect. Negative affect was defined as the average of the negative emotions (worried, rushed, irritated/angry, depressed and tense/stressed), weighted by the duration of the activities, with higher values indicating higher negative affect.

Evaluative well-being was measured by means of the Cantril Self-Anchoring Striving Scale,^24^ with steps from 0 to 10, in which 0 represents the worst possible life and 10 the best possible life.

Health status was assessed with a set of health-related questions grouped into eight health domains: vision, mobility, self-care, cognition, interpersonal activities, pain and discomfort, sleep and energy, and affect.^25^ For each question, the responses were recorded on a 5-point scale ranging from 1 (no difficulty/problem) to 5 (extreme difficulty/inability). We obtained a global health status score from the responses using a Rasch model.^26^ The health status score was then transformed into a scale ranging from 0 to 100, with higher scores representing better health status.

The presence of a depressive episode was assessed by asking whether the person had been diagnosed with depression and had been receiving treatment during the previous 12 months. Additionally an algorithm based on *The ICD-10 classification of mental and behavioural disorders: diagnostic criteria for research*,^27^ employing a set of questions based on the World Mental Health Survey Composite International Diagnostic Interview,^28^ was used to include non-diagnosed cases.

Cognitive functioning was assessed by evaluating verbal fluency with the animal-naming technique^29^ and immediate and delayed verbal recall was assessed with the Consortium to Establish a Registry for Alzheimer Disease Word List Memory.^29^ Short-term memory was assessed with digit span backward and forward tests from the Weschler Adult Intelligence Scale.^30^ A factor analysis was employed to confirm that verbal fluency, immediate verbal recall, delayed verbal recall, digit span backward and digit span forward represented one dimension. Then, we calculated a global score for cognitive functioning as the average of the *z*-scores on each of the five variables. We transformed this score into the percentile scale, with higher scores indicating better cognitive functioning.^31^

Participants were also asked to provide sociodemographic information, including age, sex, marital status, residential setting, household income, number of years of education and occupational status. We used the International Standard Classification of Occupations of the International Labour Organization,^32^ to code occupational status into nine subgroups, ranging from 1 (managers) to 9 ( elementary occupations). For the descriptive analyses, we categorized the nine categories into three levels according to the skill level. Ethical approvals from the Ethics Review Committee, National Public Health Institute, Helsinki, Finland; the Bioethical Committee, Jagiellonian University, Krakow, Poland; Ethics Review Committee, Parc Sanitari Sant Joan de Déu, Barcelona, Spain; Ethics Review Committee, La Princesa University Hospital, Madrid, Spain were obtained. Informed consent from each participant was also obtained. This study commenced in 2009, before requirements for review of all WHO-supported research by the WHO research ethics review committee had been fully implemented.

### Statistical analysis

All data were weighted to account for sampling design in each country and to generalize the study sample to the reference population. Normalized and post-stratified weights for two age groups, 18–49 and 50+ years, were used. Post-stratification corrections were made to the weights to adjust for the population distribution according to the national statistical institute’s census from each country; and for people randomly selected to participate in the survey but who did not finally participate.^33^ We calculated mean score estimates on positive affect, negative affect, evaluative well-being, health status and cognitive functioning using the direct method of age standardization to the European standard population.^34^

We analysed differences in demographics, well-being and health status and cognitive functioning across countries using the *χ^2^* test and the analysis of variance (ANOVA) test, using Bonferroni's correction for pairwise comparisons across countries. When differences were significant, Cramer’s *V*, Cohen’s *f* and Hedges’ *g* were reported as effect size measures, for *χ^2^* tests, ANOVA tests and pairwise comparisons, respectively. Cohen’s guidelines^35^ were used as a standard to evaluate the magnitude of the effect size.

To determine the correlation between health status and the three different components of well-being, we conducted ordinary least squares regression analyses: one for positive affect, one for negative affect; and a third for evaluative well-being. Sociodemographic variables, the presence of a depressive episode, cognitive functioning and country were introduced as covariates to control for potential confounders. Robust standard errors were estimated using the Taylor series linearization method^36^ to adjust for the effects of weighting and clustering. The *β* coefficients were used to assess which variables have the highest association with the outcome variable, since *β* coefficients can be employed as effect size measures in regression models. The effect of health status in each well-being variable was also assessed separately for each quintile of household income, controlling for the rest of covariates employed in the previous models.

We used a structural equation model framework to examine the possible predictors of well-being that could be included in a multiple indicators/multiple causes model, accounting for relevant demographic and clinical covariates. The latent variable well-being was constructed from experienced well-being and evaluative well-being. The maximum likelihood estimator with robust standard errors was employed. This analysis was carried out on the entire sample and for each country separately. Non-standardized (*B*) and standardized (*β*) coefficients represented the effect of health status and the other covariates in the well-being construct, and the effect size associated to each coefficient, respectively. *R^2^* measured the relationship between each of the construct’s three variables and the well-being construct.

Finally, we employed a multiple indicators/multiple causes model to examine the correlation between health and well-being, controlling for the covariates that were found to be significant in at least one of the previous structural equation models. Country was included as a covariate in the multiple indicators/multiple causes model. We chose the reference category according to the mean scores in positive and negative affect. The multiple indicators/multiple causes model fit was assessed by means of the following measures:^37^^,^^38^ (i) comparative fit index (CFI) > 0.90, indicating an acceptable fit; (ii) Tucker-Lewis index (TLI) > 0.90 indicating an acceptable fit; and (iii) root mean square error of approximation (RMSEA) < 0.08 (indicating an acceptable model fit) and < 0.05 (indicating a good fit).^39^

We performed data analysis using Mplus software, version 6 (Muthén and Muthén, Los Angeles, United States of America), for structural equation models and Stata, version 11.0 (Stata Corporation, College Station, USA), for the remaining analyses. Stata’s survey command (svy), which fits statistical models for complex survey data, was employed. For hypothesis tests, 95% confidence intervals (CI) were generated.

## Results

Table 1 shows the sociodemographic characteristics of the sample in each country. In general, differences in sociodemographic characteristics across countries were statistically significant in both age groups, but the effect sizes associated with these differences were small.

**Table 1:** Sociodemographic characteristics of the population sampled in the household survey, Finland, Poland and Spain, 2011–2012

Characteristics	18–49 years					50+ years			
	Finland (*n* = 485)	Poland (*n* = 1042)	Spain (*n* = 962)	Effect size^a^		Finland (*n* = 1491)	Poland (*n* = 3029)	Spain (*n* = 3791)	Effect size^a^
**Sex, no. (%)**				NS					0.06
Female	276 (56.91)	609 (58.45)	526 (54.68)			859 (57.61)	1844 (60.88)	2076 (54.76)	
Male	209 (43.09)	433 (41.55)	436 (45.32)			632 (42.39)	1185 (39.12)	1715 (45.24)	
**Age, mean (SD)**	37.08 (8.79)	32.55 (8.97)	35.91 (8.91)	0.21		66.49 (10.87)	66.25 (11.27)	66.66 (10.92)	0.02
**Current marital status, no. (%)**				0.05					0.06
Not in a partnership	194 (40.25)	490 (47.02)	443 (46.05)			530 (36.53)	1322 (43.64)	1465 (38.64)	
In a partnership	288 (59.75)	552 (52.98)	519 (53.95)			921 (63.47)	1707 (56.36)	2326 (61.36)	
**Residential setting, no. (%)**				0.31					0.31
Rural	90 (18.56)	460 (44.15)	140 (14.55)			348 (23.34)	1312 (43.31)	518 (13.66)	
Urban	395 (81.44)	582 (55.85)	822 (85.45)			1143 (76.66)	1717 (56.69)	3273 (86.34)	
**Occupational status, no. (%)**				0.17					0.11
Highest skill level	248 (53.91)	282 (36.15)	218 (25.98)			582 (40.33)	738 (30.85)	683 (24.40)	
Medium skill level	161 (35.00)	432 (55.38)	476 (56.73)			712 (49.34)	1324 (55.35)	1568 (56.02)	
Lowest skill level	51 (11.09)	66 (8.46)	145 (17.28)			149 (10.33)	330 (13.80)	548 (19.58)	
**Years of education, mean (SD)**	15.01 (3.25)	13.99 (3.24)	14.51 (5.25)	0.09		11.30 (4.14)	10.92 (3.67)	9.84 (6.16)	0.12
**Quintile of income, no. (%)**				0.12					0.08
First (Lowest)	80 (16.67)	250 (24.27)	185 (21.31)			346 (23.47)	900 (30.92)	686 (20.99)	
Second	38 (7.92)	132 (12.82)	110 (12.67)			347 (23.54)	552 (18.96)	694 (21.24)	
Third	73 (15.21)	124 (12.04)	160 (18.43)			303 (20.56)	498 (17.11)	715 (21.88)	
Fourth	178 (37.08)	218 (21.17)	217 (25.00)			300 (20.35)	571 (19.62)	745 (22.80)	
Fifth (Highest)	111 (23.13)	306 (29.71)	196 (22.58)			178 (12.08)	390 (13.40)	428 (13.10)	

NS: not significant; SD: standard deviation.

^a^ For categorical variables and quantitative variables effect sizes across countries were estimated using Cramer's *V* for χ^2^ test and Cohen's *f* for ANOVA test, respectively. Effect size was reported for all the differences that were found to be significant at the 95% confidence interval. Cramer´s *V* values of 0.10, 0.30 and 0.50 constitute small, medium and large effect sizes, whereas these values are 0.10, 0.25 and 0.40, respectively, for Cohen's *f.*

Note: For some variables the absolute numbers do not equal the total respondents due to missing values.

There were significant differences in positive and negative affect, evaluative well-being, health status and cognitive functioning across countries in each age group. Pairwise comparisons between countries indicated better evaluative well-being in Finland. Significant differences in positive and negative affect across countries had an effect size between small and moderate (Table 2).

**Table 2:** Estimates of well-being and health in Finland, Poland and Spain, 2011–2012

Variable	Mean score (95% CI)				Effect size (Hedges' *g*)^a^		
	Finland	Poland	Spain		Finland–Poland	Finland–Spain	Poland–Spain
**18–49 years**							
Positive affect	4.31 (4.18 to 4.44)	4.27 (4.11 to 4.43)	4.83 (4.75 to 4.91)		NS	0.52	0.38
Negative affect	0.58 (0.51 to 0.66)	0.45 (0.38 to 0.52)	0.67 (0.61 to 0.74)		0.16	NS	0.25
Evaluative well-being	7.81 (7.66 to 7.95)	6.43 (6.29 to 6.58)	6.95 (6.84 to 7.06)		0.90	0.55	0.32
Health status	74.80 (73.65 to 75.95)	71.52 (70.52 to 72.52)	75.55 (74.72 to 76.37)		0.28	NS	0.34
Cognitive functioning	66.37 (65.44 to 67.29)	58.72 (57.77 to 59.66)	58.92 (58.09 to 59.75)		0.69	0.68	NS
**50+ years**							
Positive affect	4.93 (4.86 to 5.00)	4.41 (4.33 to 4.49)	4.90 (4.85 to 4.94)		0.35	NS	0.36
Negative affect	0.26 (0.22 to 0.29)	0.51 (0.46 to 0.55)	0.66 (0.62 to 0.70)		0.28	0.49	0.16
Evaluative well-being	7.43 (7.35 to 7.52)	5.52 (5.43 to 5.61)	6.56 (6.48 to 6.63)		1.15	0.53	0.67
Health status	70.26 (69.71 to 70.81)	61.86 (61.30 to 62.42)	66.41 (65.95 to 66.86)		0.75	0.32	0.37
Cognitive functioning	58.46 (57.90 to 58.01)	46.69 (46.04 to 47.34)	47.38 (46.88 to 47.89)		0.91	0.89	NS

CI: confidence interval; NS: not significant.

^a^ Only effect size associated with significant differences found at a 95% CI in pairwise comparisons after Bonferroni correction are reported between indicated countries. Hedges' *g* values of 0.20, 0.50, and 0.80 constitute small, medium and large effect sizes, respectively.

Note: Data are weighted and age-standardized.

When both age groups were combined, mean scores in evaluative well-being were 7.47 (95% CI: 7.39–7.54) in Finland, 5.73 (95% CI: 5.65–5.81) in Poland, and 6.61 (95% CI: 6.55–6.67) in Spain. The mean scores for positive affect were 4.79 (95% CI: 4.73–4.86) in Finland, 4.37 (95% CI: 4.30–4.44) in Poland, and 4.90 (95% CI: 4.86–4.94) in Spain. The mean scores for negative affect were 0.32 (95% CI: 0.29–0.35) in Finland, 0.51 (95% CI: 0.47–0.54) in Poland, and 0.66 (95% CI: 0.63–0.69) in Spain.

To correlate health with well-being, we ran three regression models, considering each of the components of well-being as a dependent variable (Table 3). Health status, the presence of a depressive episode, and the cognitive functioning score were significantly associated with positive affect, negative affect and evaluative well-being. The analysis indicated that health status made the largest independent contributions to well-being.

**Table 3:** Adjusted correlation between well-being and health indicators, Finland, Poland and Spain, 2011–2012

Variable	Positive affect^a^			Negative affect^b^			Evaluative well-being^c^	
	Coefficient (95% CI)	Effect size, *β* coefficient		Coefficient (95% CI)	Effect size, *β* coefficient		Coefficient (95% CI)	Effect size, *β* coefficient
Age^d^	0.13 (0.11 to 0.16)	0.16***		−0.07 (−0.09 to −0.05)	−0.13***		0.02 (−0.02 to 0.05)	0.02
Sex (Ref. = female)	−0.03 (−0.10 to 0.05)	−0.01		0.02 (−0.03 to 0.06)	0.01		−0.12 (−0.21 to −0.04)	−0.03**
Married or in partnership (Ref. = no)	−0.04 (−0.13 to 0.03)	−0.02		−0.01 (−0.05 to 0.04)	−0.00		0.27 (0.18 to 0.36)	0.07***
Years of education	−0.03 (−0.04 to −0.02)	−0.09***		0.02 (0.01 to 0.03)	0.09***		0.01 (0.00 to 0.02)	0.03*
Residential setting (Ref. = rural)	0.03 (−0.06 to 0.12)	0.01		0.03 (−0.03 to 0.09)	0.01		−0.06 (−0.16 to 0.04)	−0.01
Occupational status	−0.01 (−0.03 to 0.01)	−0.02		−0.00 (−0.01 to 0.01)	−0.00		−0.05 (−0.07 to −0.03)	−0.07***
Income (Ref. = 1st/2nd quintile)	0.05 (−0.02 to 0.13)	0.02		−0.02 (−0.07 to 0.03)	−0.01		0.28 (0.19 to 0.37)	0.08***
Depressive episode (Ref. = no)	−0.31 (−0.44 to −0.18)	−0.07***		0.37 (0.26 to 0.48)	0.14***		−0.69 (−0.87 to −0.52)	−0.13***
Health status^d^	0.23 (0.19 to 0.27)	0.20***		−0.17 (−0.19 to −0.15)	−0.23***		0.44 (0.39 to 0.48)	0.29***
Cognitive functioning^d^	0.05 (0.01 to 0.09)	0.06**		−0.04 (−0.06 to −0.02)	−0.07***		0.09 (0.05 to 0.13)	0.08***

CI: confidence interval; *P < 0.05; **P < 0.01; ***P < 0.001.

^a^ Goodness-of-fit, adjusted *R*^2^ = 0.085.

^b^ Goodness-of-fit, adjusted *R*^2^ = 0.111.

^c^ Goodness-of-fit, adjusted *R*^2^ = 0.340.

^d^ Regression coefficient is reported in 10-point increments.

Note: Weighted data. Analyses were controlled for country.

An older age was significantly associated with higher positive affect and lower negative affect, whereas people with higher occupational status showed higher evaluative well-being. A higher income, being a woman, being married or living with a partner, and a longer period of education were all significantly associated with a better evaluative well-being. On the other hand, length of education was found to be inversely related to positive affect and directly related to negative affect (Table 3). In the analysis conducted separately for each quintile of household income, health status had a significant effect in each of the three well-being variables. Across quintiles, the *β* coefficients associated to health status ranged from 0.12 to 0.26 for positive affect, from −0.16 to −0.29 for negative affect and from 0.21 to 0.36 for evaluative well-being.

**Fig. 1:**
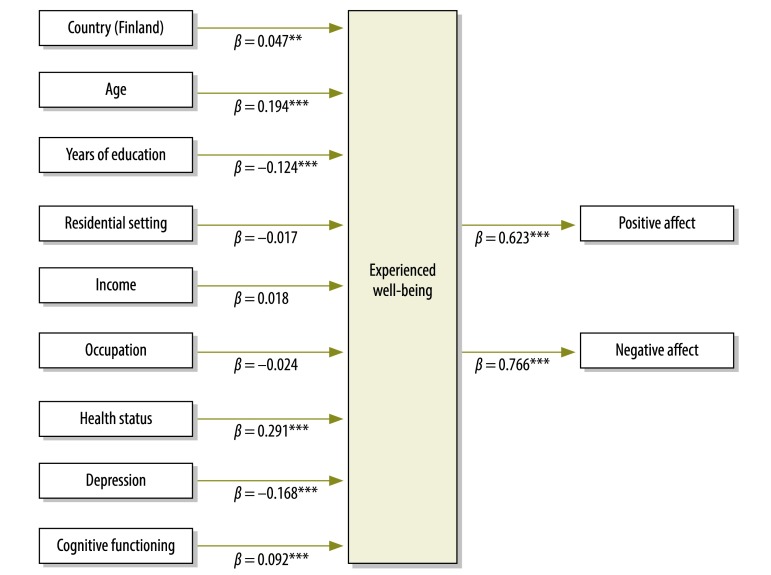
Multiple indicators/multiple causes model of relationship between health status and experienced well-being, Finland, Poland and Spain, 2011–2012

We estimated the effect of health on well-being using structural equation models for the pooled sample and separately for each country. The well-being construct comprised positive affect, negative affect and evaluative well-being. Health status and age had the strongest relationship with well-being in all samples (Table 4). Significant covariates in any of the structural equation models were included in the multiple indicators/multiple causes model shown in Fig. 1. Since evaluative well-being had a lower *R*^2^ value in the well-being construct used in the structural equation model and correlated less with positive affect (*r*: 0.24; 95% CI: 0.22–0.26) and negative affect (*r*: 0.19; 95% CI: 0.17–0.21) than positive and negative affect between themselves (*r*: 0.45; 95% CI: 0.44–0.47), evaluative well-being was excluded from the well-being construct showed in Fig. 1. Thus, the experienced well-being construct (comprising positive and negative affect) was considered as a dependent variable in the multiple indicators/multiple causes model (Fig. 1).

**Table 4:** Effect estimates of health status on well-being using structural equation models, Finland, Poland and Spain, 2011–2012

Variable	All countries				Finland				Poland				Spain		
	Effect, *B* coefficient (SE)	Effect size, *β* coefficient	*R*^2^		Effect, *B* coefficient (SE)	Effect size, *β* coefficient	*R*^2^		Effect, *B* coefficient (SE)	Effect size, *β* coefficient	*R*^2^		Effect, *B* coefficient (SE)	Effect size, *β* coefficient	*R*^2^
**Well-being**															
Evaluative well-being	1.00 (0.00)	0.43***	0.19		1.00 (0.00)	0.13**	0.02		1.00 (0.00)	0.33***	0.11		1.00 (0.00)	0.38***	0.15
Positive affect	1.11 (0.21)	0.63***	0.40		4.50 (1.52)	0.72***	0.51		1.74 (0.27)	0.61***	0.38		1.29 (0.11)	0.75***	0.57
Negative affect**^a^**	0.72 (0.14)	0.63***	0.40		2.31 (1.87)	0.73***	0.53		1.16 (0.22)	0.72***	0.51		1.06 (0.10)	0.73***	0.53
**Health status**	0.03 (0.01)	0.42***	NA		0.01 (0.00)	0.33***	NA		0.02 (0.00)	0.40***	NA		0.02 (0.00)	0.28***	NA
**Covariate^b^**															
Age	0.01 (0.00)	0.23***	NA		0.01 (0.00)	0.38***	NA		0.01 (0.00)	0.15***	NA		0.01 (0.00)	0.20***	NA
Sex	−0.03 (0.03)	−0.02	NA		−0.00 (0.01)	−0.01	NA		−0.04 (0.04)	−0.04	NA		0.03 (0.03)	0.02	NA
Married or in partnership	0.02 (0.03)	0.01	NA		0.00 (0.01)	0.01	NA		0.05 (0.04)	0.05	NA		−0.04 (0.03)	−0.03	NA
Years of education	−0.02 (0.00)	−0.13***	NA		−0.00 (0.00)	−0.09*	NA		−0.02 (0.01)	−0.10*	NA		−0.01 (0.00)	−0.12***	NA
Residential setting	0.00 (0.03)	0.00	NA		0.02 (0.02)	0.05	NA		0.04 (0.03)	0.03	NA		−0.14 (0.05)	−0.08**	NA
Occupation	−0.01 (0.01)	−0.04*	NA		−0.00 (0.00)	−0.03	NA		−0.01 (0.01)	−0.06	NA		0.00 (0.01)	0.01	NA
Income	0.07 (0.04)	0.05*	NA		−0.01 (0.02)	−0.01	NA		0.03 (0.04)	0.03	NA		0.07 (0.03)	0.05*	NA
Depression	−0.41 (0.08)	−0.17***	NA		−0.00 (0.03)	−0.01	NA		−0.40 (0.09)	−0.20***	NA		−0.34 (0.08)	−0.20***	NA
Cognitive functioning	0.01 (0.00)	0.16***	NA		−0.00 (0.00)	−0.04	NA		0.01 (0.00)	0.08*	NA		0.01 (0.00)	0.14***	NA

NA: not applicable; *R*^2^: relationship between evaluative well-being, positive affect and negative affect of the well-being construct and the whole well-being construct; SE: standard error. ; * *P* < 0.05; ** *P* < 0.01; *** *P* < 0.001.

^a^ The sign for negative affect was changed so that variables in a same construct had the same direction.

^b^ The reference category for categorical variables was the same as in the regression analysis.

Note: Weighted data. Controlled for covariates.

** *P* < 0.01; *** *P* < 0.001.

Note: Weighted data.

**Fig. 2:**
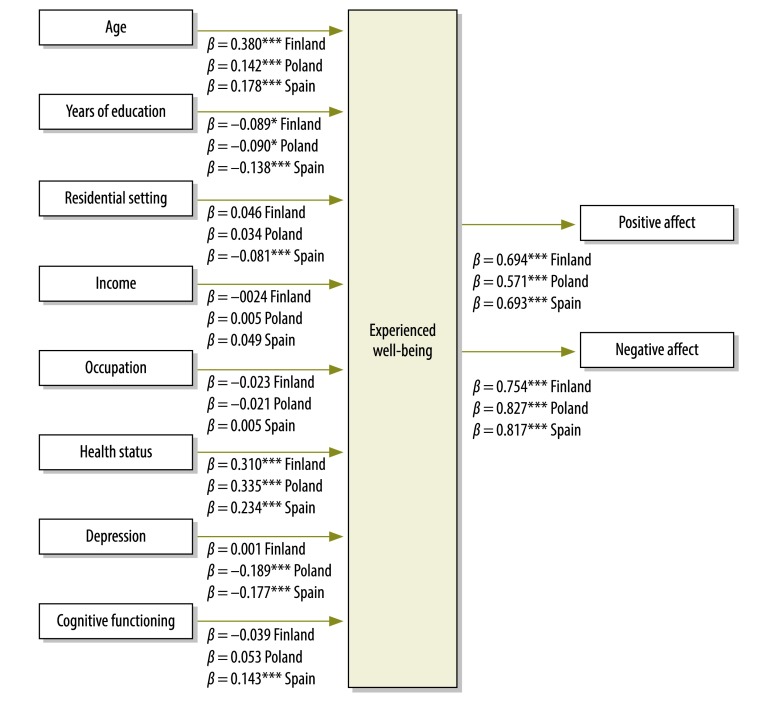
Multiple indicators/multiple causes model of relationship between health status and experienced well-being, by country, Finland, Poland and Spain, 2011–2012

The multiple indicators/multiple causes model indicated that health status had a large and significant estimated effect on experienced well-being after adjustment for potential confounding variables. Since Finland showed the best scores in positive and negative affect (Table 2), we employed a dichotomous variable (0 for living in Poland or Spain; 1 for living in Finland) in the multiple indicators/multiple causes model to control the potential effect of country. Age, cognitive functioning and the absence of depression were also related to experienced well-being, whereas length of education was found to be inversely related to experienced well-being. Moreover, people from Finland had greater experienced well-being than people from Poland and Spain. The final model presented an acceptable fit in all three measures performed (CFI: 0.95 and TLI: 0.89 RMSEA: 0.044; 90% CI: 0.037–0.050) (Fig. 1). We conducted a similar analysis for each country and found that health status, age, absence of depression (except for Finland) and lower educational level all had an estimated effect on experienced well-being in each country (Fig. 2). The results of the multiple indicators/multiple causes model for each country are similar to those of the structural equation models showed in Table 4. The multiple indicators/multiple causes model fit by country was adequate (CFI and TLI were higher than 0.90 for each country and RMSEA ranged from 0.027 to 0.033 across countries).

* *P* < 0.05; *** *P* < 0.001.

Note: Weighted data.

## Discussion

In accordance with previous studies,^14^^,^^40^ we showed that most people were quite happy and satisfied with their lives. Compared to the Gallup World Poll, which also used the Cantril Self-Anchoring Striving Scale, the three countries analysed in our study rank high on this scale.^41^ Furthermore, positive affect scores were higher and negative affect scores were lower than results from research in the USA, indicating better experienced well-being.^21^

We show that health status has the strongest relationship with all the three components of well-being even after controlling for sociodemographic variables, the presence of a depressive episode and cognitive functioning. Moreover, we show that health status has a higher association with evaluative well-being than with experienced well-being, and within experienced well-being, it has a higher association with negative affect than with positive affect. These findings can guide policy-makers to target the population at the highest risk of having poor well-being with intervention strategies aimed at improving their well-being.

Our results indicate that the evaluative component of well-being is different from the experienced components, because the statistical model that comprised positive affect, negative affect and evaluative well-being did not fit. Previous evidence has also found modest correlations between experienced and evaluative well-being.^14^ Therefore, if one’s aim is to describe a person’s well-being, a combined score of these three components should not to be created, since they are different, though interrelated, constructs. Furthermore, experienced well-being and evaluative well-being have different correlates. Our results show that higher occupational status, higher income, living with a partner and being a woman are associated with higher evaluative well-being. However, these factors do not improve the experienced well-being.^14^^,^^15^ Ageing on the other hand increases the positive affect and decreases negative affect.^14^ Although previous studies have found that evaluative well-being declines with age,^41^^,^^42^ our results showed that age did not significantly correlate with evaluative well-being, possibly because the effect of age on well-being might be explained by other variables such as health status.

This study is carried out in representative samples from different countries. It measured well-being in detail and independently from health and distinguished and captured both experienced and evaluative well-being. Nonetheless, the study’s cross-sectional design is a weakness. The results must be interpreted with caution, since causality cannot be inferred from the associations. The participation rates of this study might reflect a global decrease in response rate that has been observed in many epidemiological studies.^43^ They are similar to the ones found in other population studies carried out in Europe.^44^

In all three countries, health status correlated the strongest with well-being, even stronger than income. Most policies emphasize the importance of income on well-being,^12^ however our results show that policy-makers should favour improvement of health status to promote the well-being of the population.

Previous studies have shown an association between health and well-being in low- and middle-countries and hence our results will probably reproduce in these settings too.^45^^,^^46^ Our results show that the association between health status and well-being is also present in the people with the lowest income in the three countries. The importance of ensuring that every person achieves a basic standard of well-being is already included in the recommendations of the High-Level Panel on the Post-2015 Development Agenda.^47^

Our results indicate that clinicians should consider the well-being of the patients when developing, implementing and evaluating interventions. Furthermore, if measures of well-being are used to guide policy, both experienced well-being and evaluative well-being should be assessed. Further research might explore whether the strong association that health status has with well-being is explained by the limitations in day-to-day activities faced by people with poor health.

## References

[1] Helliwell J, Layard R, Sachs J. World happiness report. New York: The Earth Institute, Colombia University; 2012. Available from: http://www.earthinstitute.columbia.edu/sitefiles/file/Sachs%20Writing/2012/World%20Happiness%20Report.pdf [cited 2014 Mar 24].

[2] Stiglitz JE, Sen A, Fitoussi JP. Report by the Commission on the Measurement of Economic Performance and Social Progress. Paris: Commission on the Measurement of Economic Performance and Social Progress; 2009.

[3] OECD guidelines on measuring subjective well-being. Paris: Organisation for Economic Co-operation and Development; 2013.

[4] What makes us happy? Ten years of the Australian Unity Wellbeing Index. 2nd ed Melbourne: Australian Unity; 2008.

[5] How are Canadians really doing? The 2012 CIW Report. Waterloo: Canadian Index of Wellbeing; 2012.

[6] Gallup-Healthways Well-being Index. Methodology report for indexes. Washington: Gallup Inc.; 2009.

[7] Self A, Thomas J, Randall C. Measuring national well-being: life in the UK, 2012. Newport: UK Office for National Statistics; 2012.

[8] Tobgay T, Dophu U, Torres CE, Na-Bangchang K. Health and gross national happiness: review of current status in Bhutan. J Multidiscip Healthc. 2011;4:293–8. 10.2147/JMDH.S21095 21847351 PMC3155859

[9] Tobgay T, Dorji T, Pelzom D, Gibbons RV. Progress and delivery of health care in Bhutan, the land of the thunder dragon and gross national happiness. Trop Med Int Health. 2011;16(6):731–6. 10.1111/j.1365-3156.2011.02760.x 21418446

[10] Cohen S, Pressman SD. Positive affect and health. Curr Dir Psychol Sci. 2006;15(3):122–5 10.1111/j.0963-7214.2006.00420.x

[11] Lyubomirsky S, King L, Diener E. The benefits of frequent positive affect: does happiness lead to success? Psychol Bull. 2005;131(6):803–55. 10.1037/0033-2909.131.6.803 16351326

[12] Graham C. Happiness and health: lessons – and questions – for public policy. Health Aff (Millwood). 2008;27(1):72–87. 10.1377/hlthaff.27.1.72 18180481

[13] Dolan P, Lee H, King D, Metcalfe R. Valuing health directly. BMJ. 2009;339:b2577 10.1136/bmj.b2577

[14] Kahneman D, Deaton A. High income improves evaluation of life but not emotional well-being. Proc Natl Acad Sci USA. 2010;107(38):16489–93. 10.1073/pnas.1011492107 20823223 PMC2944762

[15] Kahneman D, Krueger AB, Schkade D, Schwarz N, Stone AA. Would you be happier if you were richer? A focusing illusion. Science. 2006;312(5782):1908–10. 10.1126/science.1129688 16809528

[16] COURAGE in Europe project [Internet]. Collaborative Research on Ageing in Europe; 2010. Available from: http://www.courageineurope.eu/ [cited 2013 Aug 20].

[17] Eikemo TA, Huisman M, Bambra C, Kunst AE. Health inequalities according to educational level in different welfare regimes: a comparison of 23 European countries. Sociol Health Illn. 2008;30(4):565–82. 10.1111/j.1467-9566.2007.01073.x 18298629

[18] SAGE longitudinal multi-country study [Internet]. Geneva: World Health Organization; 2014. Available from: http://www.who.int/healthinfo/sage/cohorts/en/index2.html [cited 2014 Jul 14].

[19] Process of translation and adaptation of instruments. Geneva: World Health Organization; 2013. Available from: http://www.who.int/substance_abuse/research_tools/translation/en/ [cited 2013 Aug 20].

[20] Üstün TB, Chatterji S, Mechbal A, Murray CJL. WHS Collaborating groups. Quality assurance in surveys: standards, guidelines and procedures. In: United Nations Statistics Division. Department of Economic and Social Affairs. Household sample surveys in developing and transition countries [Series F No. 96]. New York: United Nations; 2005.

[21] Kahneman D, Krueger AB, Schkade DA, Schwarz N, Stone AA. A survey method for characterizing daily life experience: the day reconstruction method. Science. 2004;306(5702):1776–80. 10.1126/science.1103572 15576620

[22] Ayuso-Mateos JL, Miret M, Caballero FF, Olaya B, Haro JM, Kowal P, et al. Multi-country evaluation of affective experience: validation of an abbreviated version of the day reconstruction method in seven countries. PLoS ONE. 2013;8(4):e61534. 10.1371/journal.pone.0061534 23626697 PMC3634002

[23] Miret M, Caballero FF, Mathur A, Naidoo N, Kowal P, Ayuso-Mateos JL, et al. Validation of a measure of subjective well-being: an abbreviated version of the day reconstruction method. PLoS ONE. 2012;7(8):e43887. 10.1371/journal.pone.0043887 22952801 PMC3428291

[24] Cantril H. The pattern of human concerns. New Brunswick: Rutgers University Press; 1965.

[25] Salomon JA, Mathers CD, Chatterji S, Sadana R, Üstün TB, Murray CJL. Quantifying individual levels of health: definitions, concepts, and measurement levels. In: Murray CJL, Evans DB, editors. Health systems performance assessment: debates, methods, empiricism. Geneva: World Health Organization; 2003 pp. 301–18.

[26] Pallant JF, Tennant A. An introduction to the Rasch measurement model: an example using the Hospital Anxiety and Depression Scale (HADS). Br J Clin Psychol. 2007;46(1):1–18. 10.1348/014466506X96931 17472198

[27] The ICD-10 classification of mental and behavioural disorders: diagnostic criteria for research. Geneva: World Health Organization; 1993.

[28] Kessler RC, Ustün TB. The World Mental Health (WMH) Survey Initiative Version of the World Health Organization (WHO) Composite International Diagnostic Interview (CIDI). Int J Methods Psychiatr Res. 2004;13(2):93–121. 10.1002/mpr.168 15297906 PMC6878592

[29] Morris JC, Heyman A, Mohs RC, Hughes JP, van Belle G, Fillenbaum G, et al. The Consortium to Establish a Registry for Alzheimer’s Disease (CERAD). Part I. Clinical and neuropsychological assessment of Alzheimer’s disease. Neurology. 1989;39(9):1159–65. 10.1212/WNL.39.9.1159 2771064

[30] The WAIS III - WMS III updated technical manual. San Antonio: The Psychological Corporation; 2002.

[31] He W, Muenchrath MN, Kowal P. Shades of gray: a cross-country study of health and well-being of the older populations in SAGE countries, 2007-2010. Washington: US Government Printing Office; 2012.

[32] International Standard Classification of Occupations ISCO-08 [Internet]. Geneva: International Labour Organization; 2012. Available from: http://www.ilo.org/public/english/bureau/stat/isco/isco08/index.htm [cited 2014 Jul 14].

[33] Moussavi S, Chatterji S, Verdes E, Tandon A, Patel V, Ustun B. Depression, chronic diseases, and decrements in health: results from the World Health Surveys. Lancet. 2007;370(9590):851–8. 10.1016/S0140-6736(07)61415-9 17826170

[34] World health statistics annual. Geneva: World Health Organization; 1990.

[35] Cohen J. Statistical power analysis for the behavioral sciences. New York: Academic Press; 1988.

[36] Wolter K. Introduction to variance estimation. New York: Springer-Verlag; 1985.

[37] Hu LT, Bentler PM. Cutoff criteria for fit indices in covariance structure analysis: Conventional criteria versus new alternatives. Struct Equ Modeling. 1999;6(1):1–55 10.1080/10705519909540118

[38] Reise SP, Widaman KF, Pugh RH. Confirmatory factor analysis and item response theory: two approaches for exploring measurement invariance. Psychol Bull. 1993;114(3):552–66. 10.1037/0033-2909.114.3.552 8272470

[39] Browne MW, Cudeck R. Single sample cross-validation indexes for covariance structures. Multivariate Behav Res. 1989;24(4):445–55 10.1207/s15327906mbr2404_4 26753509

[40] Diener E, Diener C. Most people are happy. Psychol Sci. 1996;7(3):181–5. 10.1111/j.1467-9280.1996.tb00354.x11894851

[41] Deaton A. Income, health, and well-being around the world: evidence from the Gallup World Poll. J Econ Perspect. 2008;22(2):53–72. 10.1257/jep.22.2.53 19436768 PMC2680297

[42] Stone AA, Schwartz JE, Broderick JE, Deaton A. A snapshot of the age distribution of psychological well-being in the United States. Proc Natl Acad Sci USA. 2010;107(22):9985–90. 10.1073/pnas.1003744107 20479218 PMC2890490

[43] Morton LM, Cahill J, Hartge P. Reporting participation in epidemiologic studies: a survey of practice. Am J Epidemiol. 2006;163(3):197–203. 10.1093/aje/kwj036 16339049

[44] Börsch-Supan A, Hank K, Jürges H. A new comprehensive and international view on ageing: introducing the “Survey of Health, Ageing and Retirement in Europe”. Eur J Ageing. 2005;2(4):245–53 10.1007/s10433-005-0014-9 PMC5546288 28794739

[45] Cramm JM, Møller V, Nieboer AP. Individual- and neighbourhood-level indicators of subjective well-being in a small and poor Eastern Cape township: the effect of health, social capital, marital status, and income. Soc Indic Res. 2012;105(3):581–93. 10.1007/s11205-011-9790-0 22247584 PMC3249562

[46] Temane QM, Wissing MP. The role of subjective perception of health in the dynamics of context and psychological well-being. S Afr J Psychol. 2006;36(3):564–81 10.1177/008124630603600308

[47] A new global partnership: eradicate poverty and transform economies through sustainable development. New York: United Nations; 2013.

